# Community based integrated vector management for malaria control: lessons from three years’ experience (2016–2018) in Botor-Tolay district, southwestern Ethiopia

**DOI:** 10.1186/s12889-019-7606-3

**Published:** 2019-10-21

**Authors:** Abebe Asale, Dereje Kussa, Melaku Girma, Charles Mbogo, Clifford Maina Mutero

**Affiliations:** 1 International Center of Insect Physiology and Ecology, Addis Ababa, Ethiopia; 2 College of Natural Sciences, Addis Ababa University, Addis Ababa, Ethiopia; 3 Kenya Medical Research Institute – Wellcome Trust Research Programme, Nairobi, Kenya; 4 International Center of Insect Physiology and Ecology, Nairobi, Kenya; 5 School of Health Systems and Public Health, University of Pretoria Institute for Sustainable Malaria Control, University of Pretoria, Pretoria, South Africa

**Keywords:** Integrated vector management, Malaria, Health seeking behavior, Vector control, Ethiopia

## Abstract

**Background:**

Integrated vector management (IVM) remains a key strategy in the fight against vector-borne diseases including malaria. However, impacts of the strategy should be regularly monitored based on feedback obtained through research. The objective of this study was to assess the impact of IVM for malaria control in Botor-Tolay district, southwestern Ethiopia after three years (2016–2018) of IVM implementation.

**Method:**

Prior to the implementation of IVM, a survey of socio-demographic, malaria burden, and communities’ perception towards malaria control was conducted in 200 households selected at random from 12 villages using standard questionnaire. Households were revisited after three years of project implementation for impact assessment. Compiled malaria case data was obtained from district health bureau for the three years period of the study while adult mosquito collection was conducted during each year using CDC light traps. Monthly larval mosquito collections were made each year using standard dipping method. Community education and mobilization (CEM) was made through different community-based structures.

**Results:**

The proportion of respondents who sought treatment in health facilities showed a significant increase from 76% in 2015 to 90% in 2018(*P* < 0.001). An average of 6.3 working and 2.3 school days were lost per year in a household due to parents and children falling sick with malaria. Malaria costs in a household in Botor-Tolay averaged 13.3 and 4.5 USD per episode for medical treatment and transportation respectively. Significantly fewer adult mosquitoes were collected in 2018 (0.37/house/trap-night) as compared to 2015 (0.73/house/trap-night) (*P* < .001). Malaria cases significantly declined in 2018 (262) when compared to the record in 2015 (1162) (*P* < 0.001). Despite improved human behavioral changes towards mosquito and malaria control, there were many setbacks too. These include reluctance to seek treatment in a timely manner, low user compliance of LLINs and low net repairing habit.

**Conclusion:**

The coordinated implementation of community-based education, environmental management, larviciding together with main core vector control interventions in Botor-Tolay district in Southwestern Ethiopia have contributed to significant decline in malaria cases reported from health facilities. However, commitment to seeking treatment by people with clinical symptoms of malaria and to repair of damaged mosquito nets remained low.

## Background

Vector-mediated diseases continue to be a global health threat accounting for an estimated 17% of all infectious diseases and more than 700,000 deaths due to diseases such as malaria, yellow fever, human African trypanosomiasis, leishmaniasis, dengue, schistosomiasis, Chagas disease, Japanese encephalitis and onchocerciasis, and putting more than 80% of the world’s population at risk [1]. Malaria impact ranks high among all vector borne diseases with a total of 216 million cases and 445,000 deaths reported in 2016 alone, with Africa accounting for about 90% of the disease cases and deaths [2].

Vector control constitutes a key element of disease containment and the agenda for malaria elimination as envisaged by national governments together with important stakeholders including World Health Organization, Roll Back Malaria and many other non-governmental organizations [3]. Calls to reverse malaria morbidity and mortality have been ongoing during the last three decades, especially focused on the use of long-lasting insecticide-treated nets (LLINs) and indoor residual spraying as the primary vector control tools [4]. However, following apparent stagnation of malaria reduction among different countries due to technical and other challenges, interest has increasingly shifted to the use of an integrated vector management approach aimed at, among other things, slowing down the emergence and spread of vector resistance to most public health insecticides.

Integrated vector management is a participatory approach which encourages the involvement of different stakeholders to optimize the use of resources potentially available for vector control [5, 6]. Pertinent questions defining IVM include: how can we achieve higher intervention coverage and intended protection target with less money? How can the public health sector work together with other stakeholders, for instance, agriculture and urban planning sectors that directly or indirectly affect national health policies? Which interventions work effectively, when and where? IVM emphasizes on engaging stakeholders and end-users particularly mobilizing community members as part of the intervention itself so that they become central players in disease control and prevention instead of being passive receivers of government “program packages”.

Community mobilization and education together with capacity building are necessary elements of IVM which help to bring together local community members, local government bodies, religious and civil society organizations. As an example, community mobilization and education conducted in different countries as part of IVM has proved to be effective in the control of vectors of dengue [7–9] and malaria [10–12].

In Botor-Tolay district despite the high coverage of IRS and scaling up of LLINs, there was no documented information yet on the community’s perception as to whether the envisaged control and elimination programs could work effectively. The aim of this study was to assess the impact of continuous community education and mobilization (CCEM) on knowledge, attitude and practice in relation to malaria control and prevention in the context of IVM.

## Methods

### Study area and period

This study was conducted in 12 villages located in Botor-Tolay District, Jimma Zone, Oromia regional state of Southwestern Ethiopia. The study area lies about 243 km South West of Addis Ababa, the capital city of Ethiopia, approximately 8^0^21′53.4“N and 37^o^20’12.6”E. The study area lies at an altitude ranging between 1100 to 1800 m above sea level. The mean annual temperature ranges from 19 °C to 30 °C and the mean annual rainfall varies from 400 to 1500 mm/year.

The communities are mainly engaged in mixed agriculture and small-scale trade at village level. Malaria prevalence in the area is 1.65% (cross sectional prevalence survey conducted by icipe 2015). Out of 31 total “*Kebeles*” (smallest administration unit of 500 to 700 households in Ethiopia) in Botor-Tolay district 26 of them are malarious (district health office, personal communication). Peak malaria transmission occurs during the period from September to November, like other parts of Ethiopia [13]. The study was conducted in 12 villages, selected based on accessibility and proximity (10–100 M) of mosquito breeding larval habitats in relation to houses.

### Study design

The study was conducted as part of monitoring and evaluation of the implementation of CEM for the prevention and control of malaria over a three-year period from January 2016 to December 2018. The study included the following two main components described in more detail in sections below: (a) implementation of community education and vector control interventions; (b) surveillance and impact assessment through longitudinal monitoring of trends in indoor relative adult mosquito density, mosquito larval populations, malaria case report from local health centers. In addition, two cross-sectional socio-demographic household surveys were conducted to determine community knowledge, attitude and practice, one survey at baseline in 2015/16 and the second at the end of post-intervention period in 2018.

### Interventions

The interventions used in this study include: (a) Community Education and Mobilization **(**CEM) to promote malaria IVM; (b), larval source management through larviciding with *Bacillus thuringiensis israelensis* and environmental management, (c) use of long-lasting insecticide treated nets (LLIN’s) and indoor residual spraying (IRS). The interventions are described in more detail below:

#### Community education and mobilization (CEM)

Despite considered as key component in the policy document of the NMCP, the community education and mobilization intervention were given less emphasis in Ethiopia as stakeholders and program managers focus on IRS and LLINs. Thus, in this study more focus and supervision was given to CEM to integrate it with ongoing mainstay interventions. Reformations were made in order to maximize its efficacy. These include establishment of different community-based structures, many of which had been established and used from as early as 2013 during initiation of IVM activities in Tolay area (Girma et al., unpublished) and schools’ anti-malaria clubs. Community based IVM working structures were composed of village administrator, village health extension worker, village agricultural development agent, community elder, youth organizer and community member with their total number ranging from 4 to5. These working groups were trained with CEM and larval source management (LSM) first and then entitled to mobilize the community members. Monthly campaign was conducted by these structures in rainy season and subsequent months. As part of environmental management community and student members were involved in filling temporary water bodies, stagnant pools, roadside and farmland ditches and environmental cleaning.

A group of young people with high school diploma were recruited from communities and trained as mosquito scouts by ICIPE for malaria control field coordinating staffs. The trainees in turn trained community-based malaria control working groups including various community groups and school anti-malarial club member students. ICIPE for malaria control field coordinating staff was responsible for designing, training, implementing and surveillance of both entomological and epidemiological information.

#### Vector control methods

##### Indoor residual spray (IRS)

The national malaria control program discontinued intervention of DDT in 2010 due to insecticide resistance and environmental pollution concerns [14]. DDT was first replaced by pyrethroids and following the report of widespread resistance against pyrethroids, the ministry decided in 2012 to adopt mosaic spraying approach in which different classes of insecticides are applied to different areas of the country considering the insecticide resistance information of the area [14]. Eighty-three (83%), 75 and 69% of residential structures in the district were sprayed by the Ministry of Health (MOH) as part of National Malaria Control Program (NMCP) in 2016, 2017 and 2018 respectively. The continues decline in IRS coverage was justified by 1) malaria incidence decline in the district documented in recent years 2) sustained IRS logistic constraint and 3) prioritization of more burdened districts surrounding the area. Spraying was conducted each year during the month of August to September by trained spray workers supervised by government health extension workers. Bendiocarb 80% WP was used at the rate 0.4 g/m^2^ before and during the trial period in all villages selected for IRS.

##### Long-lasting insecticidal nets (LLINs)

These were also provided by MOH as part of national malaria control activities by the NMCP. Bed net coverage of the community was 83% in 2015 (icipe impact assessment unit report, 20,115). PermaNet 2.0, (Vestergaard Frandsen Group) and DuraNet® (Shobikaa Impex pvt Ltd., Karur, Tamil Nadu 639,006, India) were the two brands of LLINs distributed by the NMCP, at the rate of one bed net for two people (personal communication to the district health office) as per WHO recommended universal net coverage.

##### Larval source management involving larviciding with Bti and environmental management

Larviciding with B*ti* and environmental management were led by ICIPE. *Bacillus thuringiensis israelensis (Bti)* granule (VectoBac G; Valent Biosciences Corporation, USA) was applied once every other week in all accessible anopheline mosquito-breeding habitats identified following standard procedure (Valent BioSciences, 2003) [15]. Up on identification of positive habitats, mosquito scouts trained with *Bti* larviciding took the measurement of the water body (length, width and depth). Then they determined the volume of water present in the breeding ground. Then Selected larval breeding habitats were treated with 3 granules/hoofprint to 125–500 g *Bti*/hectare every other week. Environmental management (EM) involved the filling or draining of all temporary mosquito breeding habitats such as hoof and footprints and small rain pools. Both EM and larviciding with *Bti* was stopped during the dry season (December, January, February, March) as malaria in Ethiopia is seasonal and there is no as such relevant breeding habitats. Larviciding then resumed during the months that follow the rainy season (April, May, June, July, August, September, October and November).

### Surveillance and impact assessment

#### Entomological monitoring

##### Larval mosquito sampling

All potential breeding habitats within the villages at the time of monitoring were inspected for larval mosquitoes using a standard dipper (11.5 cm diameter and 350 ml capacity) and pipettes following the guideline of WHO (2013) [16]. Samples were identified and categorized into *Anopheles* or *Culex* mosquito species using taxonomic key developed by Gillies & De Meillon, (1968) [17]. The location and elevation of each habitat was recorded using hand held Global Positioning System (Garmin, USA).

##### Adult mosquito sampling

Adult mosquito were sampled once in dry and wet seasons in all the 12 villages using CDC light traps (Model 512: J. W. Hock Co., Atlanta, USA). Ten CDC traps per village placed about 1.5 m from the floor near sleeping persons and ran from 8:00 p.m. in the evening to 06:00 a.m. in the morning. The ten sentinel houses per village were selected using simple random sampling and were required to be within 100 m far from each other and from mosquito breeding sites. Samples were taken from the same sentinel houses every month and every year.

##### Species identification

The collected mosquitoes were transported to nearby Tolay field *icipe*-vector biology laboratory, where mosquitoes were sorted by genus, sex and morphologically identified using taxonomic keys [18].

#### Epidemiological monitoring

Botor-Tolay district has 12 health posts and three health centers. Each village has one health posts and the three villages (Ketta, Wayu and Boro) has additional health centers each. All the 12 health posts and three health centers submit weekly malaria report to the district health office. Each health post has one to two health extension workers (HEW) trained and equipped with rapid diagnostic technique for malaria control. The HEW tested every febrile case presented to them using RDT kit and treat with Artemether/Lumefantrine (Coartem®) (all *P. falciparum* and mixed cases) and with chloroquine (all *P. vivax* cases). They refer all complicated cases to the nearby health center for further diagnosis. The three health centers are equipped with microscopy in addition to RDT. As part of the ongoing malaria cases monitoring program the trend of malaria cases was obtained from district health office. One hundred percent (100%) reporting coverage was ensured in order to get all the cases reported to respective health facilities. Data disaggregated according to population/village, total morbidity/village, age, parasite species were collected by referring to the document in the district.

#### Socio-demographic and socio-economic data collection

This survey was conducted in 200 randomly selected households distributed over 12 study villages. There is a total of 8643 households in the study villages according to district census projection report 2015 and the number of households varied from village to village with least number of households (419) documented in *Urji Oromia* and the highest number (969) was in *Ketta Bosoo village*. *Wayyu* town (the capital of the district) was excluded from this survey as it outlies from the rest of the villages with its more urban setting. Thus, households included in the study sample were proportionally drawn from each village according to their number of households. The survey was conducted using a pretested structured questionnaire which was first developed in English and then the interviewer translated into the local language (*Afaan Oromo*) during the interview time as it was stated in the questionnaire. Each data collector was trained how to translate each question included in the questionnaire. The questionnaire contained the following categories of questions: demographic characteristics; questions related to malaria transmission, causation, signs, symptoms, burden, severity of the disease, treatment-seeking behavior, local prevention and control practices. The economic impact of malaria was calculated using open ended question. Thus, respondents were requested to estimate the frequency of the episode/year, the family member get ill, the number of days lost, the amount of money spent per episode for transportation and for medical cost. All study participants were household heads with their age ≥ 18 and gave their consent to be part of the study (Additional file 1).

### Data analysis

Data was entered using SPSS version 20.00. The trend in mean malaria positivity rate reported from health facilities and mean adult mosquito population densities recorded during the study period were presented using line graphs. Chi-square test was used to determine proportional differences in respondents in relation to knowledge of malaria, malaria transmission mechanism and, control and prevention practices, before and after implementation of IVM interventions.

## Results

### Socio-demographic characteristics

In Table 1 the Socio-demographic status of Botor-Tolay community members or household heads who participated in the study was presented. There were more male-headed households than female headed households with the ratio being 4.83:1 in 2015 and 5.45:1 in 2018. According to the respondents, each household had a mean family size of 4.2 persons in 2015 and 5.5 in 2018. Roughly, half of the respondents (55.6% in 2015 and 52% in 2018) were illiterate. 32.6% of the respondents had completed primary school in 2015 and with slight improvement, the proportion of primary school completeness reached 40.5% in 2018. Farming (small-scale persistent agriculture) remains the major means of livelihood, as the overwhelming majorities (92% in 2015 and 96.5% in 2018) of the respondents were farmers. According to the respondents most of the community members (> 97%) lived in traditional houses (houses with mud plastered walls with iron sheet cover or with thatched grass cover). Most community members (83% in 2015 and 93% in 2018) kept livestock in structures which were separate from human residences.

**Table 1:** Socio-demographic characteristics before and after implementation of IVM in Botor-Tolay district southwestern Ethiopia (2015–2018)

Characteristics	Baseline (2015)	After 2018
Households sampled	200	200
Gender of head of Household (male to female ratio)	4.83	5.45
Mean family size	4.22	5.55
Mean household head age	37.5	38.96
Education n (%)		
Illiterate	111 (55.6)	105 (52.5)
Primary school	65 (32.6)	81 (40.5)
Secondary school	21 (10.7)	10 (5)
Beyond secondary school	3 (1.5)	4 (2)
Job description n(%)		
Farmer	184 (92)	193 (96.5)
Gov. employee	10 (5)	2 (1)
Private business	6 (3)	5 (2.5)
Housing condition n(%)		
Brick & cement wall, iron sheet cover	1 (0.5)	3 (1.5)
mud plastered wall, iron sheet cover	120 (60)	135 (67.5)
Mud plastered wall, thatched grass cover	79 (39.5)	62 (31)
Livestock and human quarters are separate n(%)		
Yes	166 (83)	186 (93)
No	34 (17)	14 (7)
Number of beds/sleeping spaces per family n(%)		
1	118 (59)	53 (26.5)
2	50 (25)	110 (55)
3 and more	32 (16)	37 (18.5)

### Household level awareness and knowledge about malaria transmission characteristics

Most of the respondents (85% in 2015 and 90.5% in 2018) indicated that malaria is transmitted by mosquito bites which occur during the night (Table 2). The level of awareness among respondents to mosquito biting time showed significant increase (*p* < 0.001) in 2018 when compared to 2015. Similarly, respondents showed increased level of awareness in correctly identifying mosquito breeding habitats, typical malaria symptoms and mainstay vector control interventions (LLINs and IRS) in 2018; however, some aspects of behavioral changes in awareness lack significant increment when compared to the level of awareness measured in 2015. Respondents showed significant increase (*P* < 0.001) in the level of awareness in correctly identifying peak malaria season (from 37% in 2015 to 83% in 2018), keeping the house walls without plastering for at least 6 months after spray (from 47% in 2015 to 82% in 2018) and knowing larval source management as a useful vector control tool (from 52.5% in 2015 to 88% in 2018). Discussing malaria related issues during family get together however, is not common among respondents as change in level of awareness still remains low (increased from 44.5% in 2015 to 49.5% in 2018).

**Table 2:** Assessment of knowledge related to malaria transmission characteristics, before and after implementation of IVM in Botor-Tolay district southwestern Ethiopia (2015–2018)

Variables	Attributes	Year		X^2^-square, *p*-value
		2015 n(%)	2018 n(%)	
understand mosquito bite as means of malaria transmission	Yes	170 (85)	181 (90.5)	*P* = 0.093
	No	30 (15)	19 (9.5)	
understand malaria vector mosquitoes biting time (night)	Yes	141 (70.5)	184 (92)	*P* < 0.001
	No	59 (29.5)	16 (8)	
Correctly identify potential mosquito breeding habitats (stagnant water)	Yes	161 (80.5)	170 (85)	*P* = 0.234
	No	39 (19.5)	30 (15)	
Correctly identify at least three malaria symptoms	Yes	148 (74)	154 (77)	*P* = 0.485
	No	52 (26)	46 (23)	
Identify mainstay malaria vector control interventions (IRS, LLINS)	Yes	142 (71)	150 (75)	*P* = 0.368
	No	58 (29)	50 (25)	
Malaria is treatable disease	Yes	180 (90)	178 (89)	*P* = 0.744
	No	20 (10)	22 (11)	
Correctly identify peak malaria season	Yes	74 (37)	166 (83)	*P* < 0.001
	No	126 (63)	34 (17)	
Know that walls should not be plastered for at least 6 months post-IRS	Yes	94 (47)	164 (82)	*P* < 0.001
	No	106 (53)	36 (18)	
Know that larval source management is useful vector control method	Yes	105 (52.5)	176 (88)	*P* < 0.001
	No	95 (47.5)	24 (12)	
Discuss malaria related issues during family get together	Yes	89 (44.5)	99 (49.5)	*P* = 0.316
	No	111 (55.5)	101 (50.5)	

#### Malaria testing and treatment seeking behavior

Table 3 shows malaria testing, treatment seeking behavior and estimated loses in days and money due to the disease. Trend in malaria testing and treatment seeking behavior showed significant improvement (*P* = 0.004) in 2018 with proportion of respondents who sought malaria treatment increasing from 74% in 2015 to 85.5% in 2018. Concurrently, the reported interest to seek treatment in health facilities also showed significant change (*P* < 0.001) from 76% in 2015 to 90% in 2018. Despite improved behavioral changes in seeking treatment from health facilities, the commitment among respondents to bring people with fever to health facilities within 24 h remained low, from 35% in 2015 to 42.5% in 2018. Economic data related to malaria episode (School days lost, money lost per episode for medical and transport) were not captured in 2015 and only the 2018 data was reported. Thus, an average of 6.3 working days were lost per person per year because either the parents became ill from malaria or due to time lost caring for a sick family member. Another mean 2.3 school days were lost per child per year due to malaria illness and families spent average 13.3 and 4.5 USD per episode for medical and transportation costs directly associated with malaria illness respectively.

**Table 3:** Assessment of malaria treatment seeking behavior of the communities (*n* = 200) before and after implementation of IVM in Botor-Tolay district southwestern Ethiopia (2015–2018)

Variables	Attributes	Year		X^2^-square, *p*-value
		2015	2018	
Person sought treatment	Yes	148 (74)	171 (85.5)	*P* = 0.004
	No	52 (26)	29 (14.5)	
Treatment sought from health facilities	Yes	152 (76)	180 (90)	*P* < 0.001
	No	48 (24)	20 (10)	
Treatment initiated within 24 h since the onset of first fever	Yes	70 (35)	85 (42.5)	*P* = 0.124
	No	130 (65)	115 (57.5)	
Treatment completed as per the prescription of health professional	Yes	171 (85.5)	174 (87)	*P* = 0.663
	No	29 (14.5)	26 (13)	
Work days lost due to illness and caring/year	Mean ± SE	–	6.3 ± 1.588	
School days lost due to illness/year	Mean ± SE	–	2.30 ± 0.16	
Money lost per episode (medical)	Mean ± SE	–	13.3 ± 6.04	
Money lost per episode (transportation)	Mean ± SE	–	4.5 ± 0.88	

#### Trend in malaria prevention practices in Botor-Tolay district (2015–2018)

Different malaria prevention interventions and assessment of community practices are presented in Table 4. The trend in mosquito net ownership and universal coverage (i.e. one mosquito net for every two people) showed significant improvement in 2018 as compared to the coverage documented in 2015. The proportion of households owning at least one mosquito net increased from 83.5% in 2015 to 90% in 2018 and the proportion of households attained universal coverage increased from 56.5% in 2015 to 83% in 2018. Moreover, the trend in prioritizing pregnant women and children under five when access to bed nets is limited significantly improved in 2018 (increased from 67.5% in 2015 to 89% in 2018). Despite the improved practices in ownership and coverage, people’s commitment to use already available mosquito nets in their house remained low (40% in 2015 and 49.5% in 2018). People’s knowledge and practice in repairing damaged nets and washing them according to the WHO recommendation also remained very low. In contrary to improved net coverage documented in the area, proportion of house structures sprayed in 2018 significantly dropped (from 23.5% in 2015 to 10% in 2018). Trend in communities’ participation in malaria education and larval source management showed significant improvement. Thus, the proportion of household heads participated in malaria education gatherings, community wide events increased from 33% in 2015 to 55% in 2018. Similarly, the proportion of households participating in larval source management increased from 26.5% in 2015 to 62% in 2018.

**Table 4:** Assessment of malaria prevention practices in communities (n = 200) before and after implementation of IVM in Botor-Tolay district southwestern Ethiopia (2015–2018)

Variables	Attributes	Year		X^2^-square, *p*-value
		2015 n (%)	2018 n (%)	
Household has at least one mosquito net	Yes	167 (83.5)	180 (90)	*P* = 0.055
	No	33 (16.5)	20 (10)	
Household has at least one mosquito net for every two family members	Yes	113 (56.5)	166 (83)	*P* < 0.001
	No	87 (43.5)	34 (17)	
Family members slept under mosquito net in previous night	Yes	80 (40)	99 (49.5)	*P* = 0.056
	No	120 (60)	101 (50.5)	
Priority given to pregnant woman and children under five when access is limited	Yes	135 (67.5)	178 (89)	*P* < 0.001
	No	65 (32.5)	22 (11)	
Nets repaired when there is damage	Yes	45 (22.5)	51 (25.5)	*P* = 0.482
	No	155 (77.5)	149 (74.5)	
Mosquito nets washed as per WHO recommendation	Yes	25 (12.5)	38 (19)	*P* = 0.074
	No	175 (87.5)	162 (81)	
House sprayed in the last 12 months	Yes	47 (23.5)	20 (10)	*P* < 0.001
	No	153 (76.5)	180 (90)	
House hold head ever participated in malaria education	Yes	66 (33)	110 (55)	*P* < 0.001
	No	134 (67)	90 (45)	
Household head ever participated in larval source management	Yes	53 (26.5)	124 (62)	*P* < 0.001
	No	147 (73.5)	76 (38)	

#### Malaria education, Larviciding and larval source management

Community members and school communities received education through community wide events and meetings. People trained in implementation of IVM and larval habitat management over a three-year period are presented in Table 5. In this process, more than 38,838 people (students, community members and military service men) received malaria education over three years through community wide events, coffee ceremony sessions and school-wide events. Six hundred twelve (612) people (community members, student representatives, health extension workers and policy makers) were trained in implementing IVM. In the process of involving communities in IVM, different activities including establishment of community based IVM working groups, creation of malaria-centered co-curricular activities in the schools, establishment of IVM learning centers and IVM demonstration sites were done. In an effort made to reduce potential mosquito breeding habitats, 100 ha of land was managed (drained, filled, and cleaned) through mobilization of community members, military service men and students. In addition, more than 1440 different habitats positive for mosquito larvae were treated with a total of 50 kg *Bti* on bi-weekly bass for three years.

**Table 5:** Community members involved in malaria education, involved in larval source management and breeding habitat managed in Botor-Tolay district (2016–2018)

Community members involved/habitats managed/*Bti* used	Year			Total
	2016	2017	2018	
Community members received education	6528	9325	6600	22,453
School communities received malaria education	2473	4100	6226	12,799
Schools’ anti-malaria clubs trained	40/each	Re-enforced	Re-enforced	480
Community working groups trained in each village	4	Re-enforced	Re-enforced	48
Community members participated in LSM	360	1325	488	2173
Military service people participated in LSM	1151	1233	1202	3586
Health professional and policy makers trained	28	28	28	84
Breeding habitat managed (hectare)	36	34	30	100
*Bti* applied in breeding habitats (kg)	18 kg	17 kg	15 kg	50 kg

#### Trend in adult *Anopheles* mosquito abundance

**Fig. 1:**
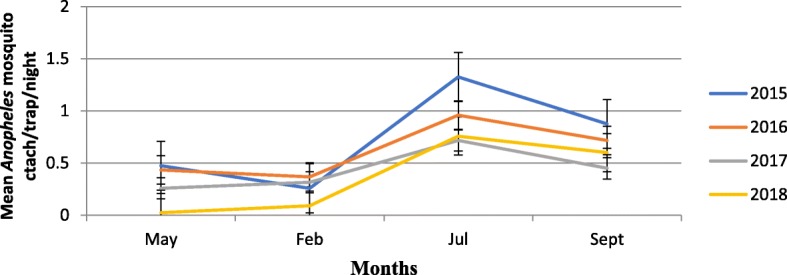
Adult Anopheles population dynamics in the study area (2015–2018)

Malaria vector population dynamics documented for four years is presented in Fig. 1. Over all 3445 anopheline and culicine mosquito were collected over four years period from 12 villages in Botor-Tolay using CDC light traps. Out of the total 3445 mosquitoes 1035 were *An. gambiae* s.l. and the rest 2410 were Culex mosquitoes. The highest total number of *Anopheles* mosquitoes (352) was collected in 2015 and the least (177) in 2018. There was no significant difference in mean mosquito density between 2015 and 2016 but significantly low mean mosquito density (*P* < 0.001) was collected in years 2017 (0.44/house/trap-night) and 2018 (0.37/house/trap-night) as compared to the mean mosquito density collected in 2015 (0.73/house/trap-night).

#### Trend in Anopheles mosquito larva abundance

**Fig. 2:**
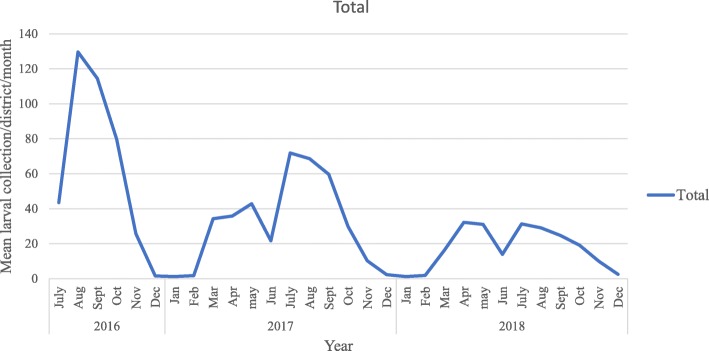
Mean total Anopheles larvae collected from 12 study villages in Botor-Tolay district (2016–2018)

Monthly Anopheles mosquito larvae collection was made since July 2016 to December 2018 and presented in Fig. 2. Over all 11, 846 anopheline larvae were collected over two and half years sampling period from 12 villages in Botor-Tolay district using standard dipping method. Out of the total 11,846 Anopheline larvae, the highest number, 4734 (40%) was collected in 2016 and the least number, 2555 (21.6%) was collected in 2018.

#### Reported malaria cases in Tolay (2015–2018)

**Fig. 3:**
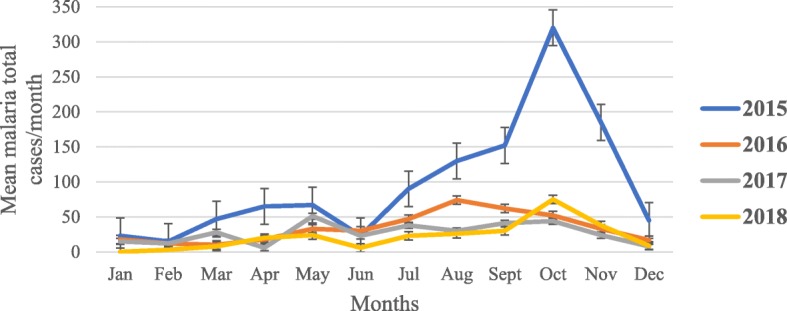
The mean malaria total cases reported from health facilities from health facilities in Botor-Tolay district Southwestern Ethiopia (2015–2018)

Figure 3 shows trend of total malaria cases reported from health facilities found across 12 villages in Botor-Tolay. A total of 2065 malaria cases were documented over the four years from the health facilities (health centers, health posts) found in 12 study villages. The highest case report (1162) was documented in 2015 and the lowest case report (262) was documented in 2018. The rest 406 and 320 cases were recorded in 2016 and 2017 respectively. Malaria cases significantly declined (*P* < 0.001) in 2016, 2017 and 2018 when compared to the base line data taken in 2015 however, further mean separation test showed that there was no statistically significant difference in mean malaria cases documented among years 2016, 2017 and 2018.

**Fig. 4:**
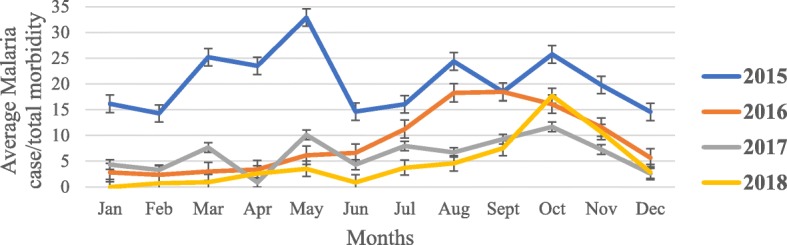
The percent of malaria cases reported from health facilities in comparison to all cause morbidity in Botor-Tolay district Southwestern Ethiopia (2015–2018)

The percent of malaria cases reported from health facilities in comparison to all cause morbidity in Botor-Tolay between 2015 and 2018 were presented in Fig. 4. Thus, the share of malaria disease burden is the highest in 2015 accounting average 20.50% of all cause morbidity. Its burden share reduced to average 6.80, 6.34 and 4.65% in 2016, 2017 and 2018, respectively.

#### The relationship between monthly malaria cases and meteorological variables

**Fig. 5:**
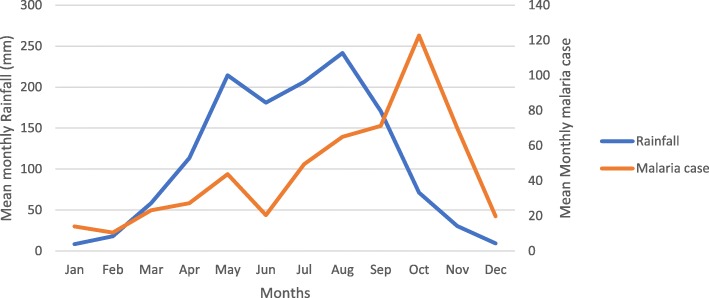
Mean monthly rainfall pattern and corresponding malaria incidence in Botor-Tolay district, South western Ethiopia (2015–2018)

The relationship between climatic factors (Rainfall, maximum temperature, minimum temperature, relative humidity) and reported malaria cases in Botor-Tolay district was shown in Table 6. Accordingly, climatic factors relative humidity (*P* < 0.001), rainfall (*P* = 0.002) and minimum temperature (*P* = 0.003) had aggravative impact on malaria morbidity showing positive relationship whereas maximum temperature showed negative relationship. In addition, the monthly rainfall pattern and its impact on monthly malaria incidence between years 2015 and 2018 was presented in Fig. 5. Thus, major rainy season starts in July and continues until the beginning of September. Intermittent raining may continue until October but the subsequent months (November to February) remain mostly dry. Short rainy season starts towards the second half of March and continues to fall until the first half of May. The second half of May and June mostly remains dry. Malaria case build ups also follow up the rainfall pattern with slight lag. The total annual rainfall measurement for the district was found to be 1406.4 mm, 1471 mm, 1231.8 mm and 1185.1 mm in 2015, 2016, 2017 and 2018 respectively.

**Table 6:** The relationship between climatic variables and malaria morbidity in Botor-Tolay district (2016–2018)

*Meteorological Variable*	*Correlation coefficient*	*p-value*
Mean Relative humidity	0.728433	< 0.001
Mean rainfall	0.496976	0.002
Mean Minimum temperature	0.484132	0.003
Mean Maximum temperature	−0.319760	0.057

## Discussion

In this study, the critical role played by the community in implementing integrated vector management for malaria control was investigated. In addition, the trend in behavioral change in community engagement in vector control activities, as well as their overall knowledge, attitude and practice towards malaria control and prevention was assessed. Year to year changes in the number of malaria cases at the local health centers and in the number of adult *Anopheles* mosquitoes collected indoors were used coarsely as the impact indicators of sustained community education and mobilization and implementation of actual mosquito control measures in the context of IVM. In this regard, an important limitation which warrants the results to be interpreted with caution was the fact that, due to ethical considerations, there were no non-intervention control villages where mosquito and malaria parameters could be measured in parallel for comparison purposes. It was also not feasible to implement a study design that could separately quantify the effects of CEM compared to those of the actual vector control methods, or from those of other confounding factors e.g., yearly variations in the occurrence and geographic extent of the potential mosquito breeding habitats due to variations in climatic variables such as rainfall.

Assessment of trends in people’s knowledge before and after implementation of IVM in Botor-Tolay district towards malaria epidemiology including the mechanism of malaria transmission, time of mosquito bite, correctly identifying peak malaria season, correctly describing clinical manifestations of malaria, identifying potential mosquito breeding habitats showed significant improvement in 2018 as compared to the assessment made back in 2015. Despite, the lack of clear policy on IVM in Ethiopia, elements of IVM such as community mobilization and education, malaria education using IEC/BCC, through mass media such as radio and TV have been implemented by the NMCP since 2006 [19]. These concerted efforts by the NMCP to raise public awareness about malaria disease are reflected in increased awareness of the public according to community-based assessments made in recent years across the country (Amhara, Tigray, Gurage, Oromia, southern nations) which showed high improvement trend in people’s knowledge on clinical symptoms [20, 21], mosquito bite as means of malaria transmission [22, 23] and stagnant water as mosquito breeding habitat [20]. In this study high level of knowledge about mainstay vector control interventions (LLINs and IRS), peak malaria season, how to safely handle IRS sprayed walls and knowing larval source management as useful vector control tool was documented. The improved knowledge level among community members clearly telling LLINs as major vector control intervention and right peak malaria season in this study were further supported by reports by Yimer et al. (2015) [22] from Abashege site and Yewhalaw et al. (2010) [24] from Asendabo area, southwestern Ethiopia. High level recognition of the role of IRS in malaria control in this study contrasted with the reports from both Yewhalaw et al. (2010) [24] and Aderaw and Gedefaw (2013) [25] whom documented very low awareness level up to 16.5 and 5.3% respectively.

In this study the trend in malaria testing and treatment seeking behavior showed significant improvement with 85.5% of the respondents seeking some form of treatment. Concurrently, the reported interest to seek treatment in health facilities also showed significant improvement with 90% of the respondents reporting that they brought people with any sign of disease to health facilities (government and private clinics). Generally, treatment seeking behavior for malaria diseases showed improvements across the country in recent reports [13;24]. Higher treatment seeking behavior by community members was reported from Jimma Zone, southwestern Ethiopia by Yewhalaw et al. (2010) [24] with 71.5% and 33.8 of the respondents seeking treatment in public and private health facilities respectively. Higher treatment seeking behavior (82%) was also recorded in nationwide study conducted in 2015 by Federal ministry of health in collaboration with Ethiopian public health institute [13]. Thus, improvements recorded in treatment seeking behavior in this study should be considered not only as a result of IVM interventions deployed but also partly because of the national malaria control programs put in place. Despite improved behavioral changes in seeking treatment from health facilities, the commitment among respondents to bring people with fever to health facilities within 24 h since the onset of clinical symptoms remains low. This is also a common challenge reported in nationwide studies conducted by federal ministry. According to malaria indicator survey conducted in 2007, 2011 and 2015, only 15.5, 51.3 and 38.4% of respondents who had children with fever in two-week time prior to the interview had brought them to health facilities within 24 h since the onset of fever respectively [13, 26, 27].

In this study it is shown that an average 6.3 working days were lost per year because either the parents get ill from malaria or due to caring members of the family. In addition to the working days, average 2.3 school days were lost per year due to malaria illness. Families spent average 13.3 and 4.5 USD per episode for medical and transportation costs directly associated with malaria illness. Studies conducted in Ethiopia estimating working or school days lost due to malaria illness are rare but a study conducted by icipe-impact assessment unit led by Minale Kassie (Kassie et al. 2015; unpublished) conducted in Tolay documented an average loss of 6.03 labor days in a fortnight, and incurred about average Birr 9.96 in malaria treatment during the same period. Another study conducted by Sicuri et al. (2013) [28] which analysed the economic costs of malaria in children under five years old in Ghana, Tanzania and Kenya confirmed the loss of approximately US$ 5 per episode per child for non-complicated malaria in Tanzania and US$ 288 per episode per child for cerebral malaria with neurological sequelae in Kenya.

Community engagement in vector control activities and disease control practices in Botor-Tolay district showed significant improvement in 2018 as compared to the coverage documented in 2015. Proportion of households owning at least one mosquito net increased to 90% and proportion of households reached universal coverage were 83%. Pregnant woman and children under five were given priority when there is net scarcity. Increased community members involvement in malaria education and environmental management including draining, filling, and cleaning was also documented. Overall improvement in access to malaria and vector control practices were documented in study conducted in Abashege area, Guraege zone, south central Ethiopia (Yimer et al. 2015) [22] where access to at least one LLINs per household reached up to 98.7%. This is a big stride towards ensuring universal coverage set by the NMCP and by far better than the 64% national average coverage of one LLIN per household according to the latest report from federal ministry [13]. Despite the improved practices in ownership and coverage, people’s commitment to use already available mosquito nets in their houses, repairing damaged nets and washing them according to the WHO recommendation also remains very low. This could be attributed to relatively short period of education which we provided (three years only) as some behavioral changes may take longer time to be reflected. In this study, proportion of house structures sprayed in 2018 significantly dropped as compared to 2015. This could be due to government prioritization of spray operation and channeling it to more transmission intense areas. Lack of compliance in proper use of the LLINs has proved to be a major problem throughout the country [13]. According to the study conducted by EPHI, only 40% of the respondents used the net in previous night. Low net maintenance rate (3.7%) was documented in study conducted by Batisso et al., (2012) [29].

Community education and mobilization (CEM) in malaria control and prevention has been long stayed tradition in Ethiopia [10]. However, harmonizing it with other mainstay vector control interventions and mainstreaming it to policy level is a long overdue. In this study we showed the essence of CEM in curbing the disease burden through directly engaging community members (farmers, youth, school communities, military service men). In the process of involving communities in implementing IVM, different activities including establishment of community based IVM working groups, creation of malaria-centered co-curricular activities in the schools led by students, establishment of IVM learning centers and IVM demonstration sites were done. More than 100 ha of land was managed (drained, filled, and cleaned) in order to interrupt mosquito breeding. Different larva positive habitats were treated with *Bti* on bi-weekly basis during larval peak season for three years. The increased engagement of communities in malaria control and prevention has led to dramatic decline in both the vector population and the disease burden. Yearly Mosquito density significantly declined in 2018 as compared to the density documented in 2015. Similarly, the measure of larvae population also showed significant decline between 2016 and 2018. As it is documented across different seasons, the vector population density dramatically declines in dry months (December, February and March) and starts to build in late March from which the population continues to grow until late May. There is brief decline in vector population between late May and June, but it starts to gain momentum again starting from July where it continues to peak until September. The rainfall pattern especially after a dry period affects how quickly a mosquito population builds up, hence the importance of when IRS is done and how effective larviciding can be. The temporal variation and seasonality of malaria vector population density in Ethiopia is well documented [30] and implementation of vector control measures were also designed in conformation with these variations [31]. We also examined the role of climatic factors, relative humidity, rainfall, minimum and maximum temperature of the area in relation to malaria morbidity. To this end the three climatic variables; relative humidity, rainfall and minimum temperature showed significant positive relationship with malaria morbidity likely contributing to the ongoing disease transmission. Temperature and rainfall are the key deriving climatic factors in malaria transmission in Ethiopia [32, 33]. The timing of indoor spray operations which is often conducted in August and September in Ethiopia would however be much effective if done taking in to account the dynamics of local climatic variables. This is because the onset, the intensity and the duration of the rainy season varies according to the local or reginal setting. This is evidenced for instance in Amhara region [34] where malaria burden stands higher in months of April and May following the onset of short rainy season which is in contrast to the reports from the rest of the country [35–37] where higher malaria burden is often documented in September and October. Moreover, spray operations are conducted once per year which is understandably due to the expensive nature of IRS intervention but there should be re-visit to cost benefit analysis made decades ago under different climatic and policy settings. Thus, targeted IRS operations conducted twice per year would not only curb the case build ups that we observe following the short and long rainy seasons but also it will significantly reduce the vector population which carries over the parasite to the next season.

Concurrently, malaria cases reported from health facilities also significantly declined across all the study villages. As we have described earlier; the documented reduction in cases in the study area were not solely attributed to the IVM activities introduced by this study group however increased engagement of communities through integrated vector management strategy has been found effective in significantly reducing malaria disease in Zambia [38], Tanzania [39] and Nigeria [40]. According to WHO position statement [41] Integrated vector management strategies including CEM, integration of both chemical and non-chemical methods, intra- and inter-sectoral collaboration, capacity building, evaluation of the whole process to make informed decision and supporting it with appropriate legislation not only helps us to achieve the envisaged goal with less expenditure but it is also an environmentally safe approach.

## Conclusion

The introduction of IVM in Botor-Tolay district in Southwestern Ethiopia has led to significant decline in both malaria cases reported from health facilities and vector population documented during the study period. People’s knowledge and practice of malaria prevention and control methods have significantly improved following the implementation of IVM in Botor-Tolay district, however the commitment among respondents to bring people with fever to health facilities within WHO recommended 24 h since the onset of clinical symptoms is very low. In addition, the practice of washing mosquito nets following WHO procedures and repairing them when there is damage is very low. In this study the incidence of malaria showed largely a bi-modal peak between March and May (the highest) and between September and November (the second peak) thus, spray operations would be much effective if done taking in to account the onset of these peaks preferably late march and late June/July.

## Supplementary information

**Additional file 1.** Cross sectional Survey Questionnaire.

## Data Availability

The datasets used and/or analysed during the current study are available from the corresponding author on reasonable request.

## Abbreviations

ACT: Artemisinin-based Combination Therapy
BCC: Behavioral Change Communication
*Bti*: *Bacillus thuringiensis israelensis*
CDC: Center for Disease Control and Prevention
CEM: Community Education and Mobilization
DDT: *Dichlorodiphenyltrichloroethane*
IEC: Information Education and Communication
IRS: Indoor Residual Spray
ITN: Insecticide Treated Net
IVM: Integrated Vector Management
LLINs: Long-lasting Insecticidal Nets
NMCP: National Malaria Control Program
WHO: World Health Organization
