# Supplementary material for: Community based integrated vector management for malaria control: lessons from three years’ experience (2016–2018) in Botor-Tolay district, southwestern Ethiopia
Source: BMC Public Health. 2019 Oct 21;19:1318. doi: 10.1186/s12889-019-7606-3 (PMC6805624; doi:10.1186/s12889-019-7606-3)
Supplement: Supplementary file 1 — Additional file 1. Cross sectional Survey Questionnaire. [file 12889_2019_7606_MOESM1_ESM.doc]

**International center for Insect Physiology and Ecology (*icipe*)**

**Addis Ababa**

**Integrated vector management unit**

**Consent Form for field data collection**

**Part A: Information Sheet**

***Name of the Principal Investigator****: Abebe Asale* (Dr.)

***Name of the organization****:* **International center for Insect Physiology and Ecology (*icipe*)**

**Prepared by:** This consent form sheet is prepared by *Dr. Abebe Asale* whose main aim is to study The investigator is a staff member in **International center** *Community based integrated vector management for malaria control: Lessons from three years’ experience (2016-2018) in Botor-Tolay district, Southwestern Ethiopia.*

**Background**

Malaria control programs are mainly operated by the federal ministry of health and the regional health bureau with minimal participation of communities and other non-governmental organizations. Malaria control awareness and community empowerment in larval control are lacking in many malarious regions of Ethiopia.

One of the objectives of the IVM project (2016-2018) was to promote adoption and sustainability of IVM at community level in Botro-Tolay district Southwestern Ethiopia. Under this objective, community based IVM working groups and School anti-malaria clubs have been established. In addition, different community wide events including larval source management, education, school events such as awareness creation gatherings and celebrations through drama have been conducted to take the malaria control and prevention agenda directly in to the community.

## Therefore; the objective of this survey is; (1)To assess the change in knowledge, attitude and practically gained experiences in combating and preventing malaria disease during the project implementation time. 2) To assess the trend of malaria disease burden in communities in aforementioned years.

***Procedure:***

We kindly invite you to take part in this project, which is aimed at assessing the change in knowledge, attitude and practically gained experiences in combating and preventing malaria disease during the project implementation time and determining the trend of malaria disease burden in communities in aforementioned years. If you are willing to participate in this project, you need to understand and sign the agreement form.

**Site of data collection:** Botor-Tolay district

We will consider you one of our study participants in this survey. We will ask you questions related to malaria diseases transmission, control and prevention. We will ask you questions related to your family, your educational and occupational status.

**Risk and Discomfort**

There is no risk of any infection as we do this study but we may take some 45 min to 1 hour from your working time to interview you.

***Benefits***

Your participation will help us in *determining effectiveness of integrated vector man management in controlling and preventing malaria disease*, which will be an important information for policy decision making.

***Incentives***

You will not be provided any incentives to take part in this research.

***Confidentiality:***

The information that we collect from this research project will be kept confidential. Information about you that will be collected from the study will be stored in a file, which will not have your name on it, but a code number assigned to it. It will be kept under lock and key, and it will not be revealed to anyone except the principal investigator and the concerned health professional.

***Right to refuse or withdraw***

You have full right to refuse from participating in this research if you do not wish to participate or to quit at any time; and this will not compromise the health services you get at the health institutions in any way at any time.

***Whom to contact***

If you have any questions you can contact any of the following two individuals and you may ask at any time you want:

Principal Investigator: ***Dr. Abebe Asale*, *icipe***

Telephone no:+*251912123305*

OR

***Dereje Kussa*, *icipe***

Tel: +*251911830568*

*OR*

*Prof. Clifford Mutero icipe, Kenya*

*Tel:+254704835177*

This study was conducted after getting approval from Kenya Medical Research Institute (KEMRI) Scientific and Ethics Review Unit.

## Part-I : Household questionnaire

Kebele: ____________________________ Name of HH head: ______________________________ ______Date ________________

| I. **Socio-demographic characteristics** | | Attributes | | | | | | | | | |
| --- | --- | --- | --- | --- | --- | --- | --- | --- | --- | --- | --- |
| **Variables** | | 1 | 2 | 3 | 4 | 5 | 6 | 7 | 8 | 9 | 10 |
| 1 | Sex (1 = male, 2 = female) |  |  |  |  |  |  |  |  |  |  |
| 2 | Family size _________________ |  |  |  |  |  |  |  |  |  |  |
| 3 | Age (in completed years) _____________ |  |  |  |  |  |  |  |  |  |  |
| 4 | Educational status (1 = Illiterate, 2 = read and write, 3 = primary education, 4 = secondary Educ, 5 = beyond secondary educ.) |  |  |  |  |  |  |  |  |  |  |
| 5 | Occupation (1 = Farming, 2 = private business, 3 = government employee, 4 = daily laborer, 5 = other) |  |  |  |  |  |  |  |  |  |  |
| 6 | **Socio-economic conditions** |  |  |  |  |  |  |  |  |  |  |
| 7 | ***Type of house*** (1 =Brick and cement wall 2 = Corrugated Iron, 3 = Thatched roof) |  |  |  |  |  |  |  |  |  |  |
| 8 | ***Houses protected with mosquito screen (1=yes, 2=no)*** |  |  |  |  |  |  |  |  |  |  |
| 9 | ***Number of sleeping rooms ______________*** |  |  |  |  |  |  |  |  |  |  |
| 10 | ***Do you have separate animal house/shed (1 =yes, 2 = no)*** |  |  |  |  |  |  |  |  |  |  |

| I. **Socio-economic characteristics** | | Attributes | | | | | | | | | |
| --- | --- | --- | --- | --- | --- | --- | --- | --- | --- | --- | --- |
| **Variables** | | 1 | 2 | 3 | 4 | 5 | 6 | 7 | 8 | 9 | 10 |
|  | ***Respondents knowledge about malaria*** |  |  |  |  |  |  |  |  |  |  |
| 11 | ***Have you heard of malaria?*** (1 = yes, 2 = no) |  |  |  |  |  |  |  |  |  |  |
| 12 | ***From which source have you heard for the 1^st^ time?*** (1 = HEW, 2 = family member, 3 = mass media, 4 = school, 5 = church/mosque, 6 *= icipe*, 7=other specify_________________) |  |  |  |  |  |  |  |  |  |  |
| 13 | ***What causes malaria?*** (1 = Mosquito bite, 2 = Exposure to sun, 3 = cold weather, 4 = Witchcraft, 5 = bad sprit, 6 = breastmilk, 7 = shaking hands of malaria patient, 8 = eating raw maize, 9 = work load, 10 = other) |  |  |  |  |  |  |  |  |  |  |
| 14 | ***Do you know that malaria can be transmitted*** (1 = yes, 2 = no) |  |  |  |  |  |  |  |  |  |  |
| 15 | ***What is the usual time for mosquito bite?*** (1 = night, 2 = day, 3 = always, 4 =I don’t know) |  |  |  |  |  |  |  |  |  |  |
| 16 | ***Where do mosquitoes do breed?*** (1 = stagnant water, 2 = ditch, 3 = hoof print, 4 = swampy areas, 5 = pond, 6 = dirty place, 7 = cattle shed, 8 = tall grass, 9 = latrine, 10 = other) |  |  |  |  |  |  |  |  |  |  |
| 17 | ***In which season do you think high malaria transmission occurs?*** (1= Sept-Nov 2 = DEC-Feb, 3 = March-May, 4 = June-Aug) |  |  |  |  |  |  |  |  |  |  |

|  | ***Respondents knowledge about malaria*** | Attributes | | | | | | | | | |
| --- | --- | --- | --- | --- | --- | --- | --- | --- | --- | --- | --- |
|  |  | 1 | 2 | 3 | 4 | 5 | 6 | 7 | 8 | 9 | 10 |
| 18 | ***What are common malaria symptoms?*** (1 = Fever/hot body, 2 = Shivering, 3 = Weakness, 4 = Muscle Pain/Joint pain, 5 = Headache, 6 = loss of appetite, 7 = Shivering, 8 = Thirsty, 9 = Chills, 10 = vomiting, 11= others) |  |  |  |  |  |  |  |  |  |  |
| 19 | ***Which methods are used to prevent malaria disease?*** (1=sleeping under net, 2= wearing long sleeved cloths 3= fire & smoke, 4= spraying insecticide, 5= cleaning the environment, 6= screening windows & doors, 7= I don’t know) |  |  |  |  |  |  |  |  |  |  |
| 20 | ***Does your household have any mosquito nets that can be used while sleeping?*** (1 = yes, 2 = no) |  |  |  |  |  |  |  |  |  |  |
| 21 | ***How many mosquito nets does your house have? ______________*** |  |  |  |  |  |  |  |  |  |  |
| 22 | ***Observe all nets and rate the general condition.*** (1 = GOOD (NO HOLES), 2 = FAIR (small holes that do not fit a torch battery), 3= POOR (1-4 holes that fit a torch battery), 4= UNSAFE (>5 holes that fit a torch battery), 5=UNUSED (still in package) |  |  |  |  |  |  |  |  |  |  |
| 23 | ***How long ago (in months) did your household obtain the mosquito net?______*** |  |  |  |  |  |  |  |  |  |  |
| 24 | ***Observe and record the net brand*** (1= Permanet, 2= Olyset, 3= MagNet, 4 =Interceptor, 5=Yorkool, 6= Dawanet, 7 =DuraNet, 8= other specify) |  |  |  |  |  |  |  |  |  |  |
| 25 | ***Universal net coverage (1 net per 2 people) is achieved. (1=yes, 2=no)*** |  |  |  |  |  |  |  |  |  |  |
| 26 | ***Did your house get sprayed over the last 12 months? (1=Yes, 2=no)*** |  |  |  |  |  |  |  |  |  |  |
| 27 | ***Have you ever attended malaria education?*** (1=yes, 2= no) |  |  |  |  |  |  |  |  |  |  |
| 28 | ***Have you ever participated in larval source management?*** (1=yes, 2=no) |  |  |  |  |  |  |  |  |  |  |

|  | **Treatment and Health seeking behavior** | Attributes | | | | | | | | | |
| --- | --- | --- | --- | --- | --- | --- | --- | --- | --- | --- | --- |
|  |  | 1 | 2 | 3 | 4 | 5 | 6 | 7 | 8 | 9 | 10 |
| 29 | Is there anyone in your household who got seek in the last 12 months due to malaria? (1=yes, 2=no, ***if no skip to No45***) |  |  |  |  |  |  |  |  |  |  |
| 30 | ***If yes, Who was sick due to malaria in the last 12 months****?* (1 = children u5, 2 = children 5- 10, 3 = 11-15, 4 = more than 15 years, 5 = husband, 6 = wife, 7 = relative, 8 = home/farm assistant, 9 = other) |  |  |  |  |  |  |  |  |  |  |
| 31 | ***If you were sick due to malaria How many days were lost? ____________*** |  |  |  |  |  |  |  |  |  |  |
| 32 | ***If your children were sick due to malaria How many days were lost due to caring? _______________*** |  |  |  |  |  |  |  |  |  |  |
| 33 | ***If the children sick was student, how many school days were lost? ________*** |  |  |  |  |  |  |  |  |  |  |
| 34 | ***Did the person seek treatment? (1=yes, 2=no)*** |  |  |  |  |  |  |  |  |  |  |
| 35 | ***If the person did not seek treatment what was the reason?*** (1=lack of money, 2=health center is far, 3=person was not seriously ill, 4= others) |  |  |  |  |  |  |  |  |  |  |
| 36 | ***If yes when did the person start the treatment*** *(1=within 24 hours, 2= after one day, 3=after two day, 4=after three days, 5= others)* |  |  |  |  |  |  |  |  |  |  |
| 37 | ***Where did you get the treatment?*** (1=health post, 2=government health center, 3=private clinic, 4=self-medication, 5=local healer, 6=religious healer, 7=others) |  |  |  |  |  |  |  |  |  |  |
| 38 | ***Did you ever share drug with your neighbor/family member? (1=yes, 2=no)*** |  |  |  |  |  |  |  |  |  |  |
| 39 | ***Did the person finish the anti-malarial drugs***? (1=yes, 2=no) |  |  |  |  |  |  |  |  |  |  |

|  | **Treatment and Health seeking behavior** | Attributes | | | | | | | | | | |
| --- | --- | --- | --- | --- | --- | --- | --- | --- | --- | --- | --- | --- |
|  |  | 1 | 2 | 3 | 4 | 5 | 6 | 7 | 8 | 9 | 10 | |
| 40 | ***How much did you pay for the treatment per single episode per a person?*** |  |  |  |  |  |  |  |  |  |  | |
| 41 | ***How much did you pay for the transport per single episode per a person? _*** |  |  |  |  |  |  |  |  |  |  | |
| 42 | ***Do the household have capacity to afford the cost of the treat?*** (1= yes, 2= no) |  |  |  |  |  |  |  |  |  |  | |
| 43 | ***Who pay for the health service and related costs?*** (1=freely accessible, 2= sold assets, 3=lend money, 4=from relatives, 5= from saved money, 6=others) |  |  |  |  |  |  |  |  |  |  | |
| 44 | ***Who was responsible for giving care or taking a sick person to health settings?*** (1= mother, 2=father, 3=other) |  |  |  |  |  |  |  |  |  |  | |
| 45 | ***What methods do you use to prevent yourself/family from mosquito bite? (more than one answer is possible (1=I don’t use any method, 2=bed net, 3=wall spray, 3=house screening, 4=repellent, 5=others (specify____________)*** |  |  |  |  |  |  |  |  |  | |  |
| 46 | ***Do you sleep under bed nets regularly?*** (1=yes, 2=no) |  |  |  |  |  |  |  |  |  | |  |
| 47 | ***Did you sleep under bed net last night?*** (1= yes, 2= no) |  |  |  |  |  |  |  |  |  | |  |
| 48 | ***If no, what was your reason to not sleep under bed net? (****1= it irritates me, 2= there is no mosquito & no malaria, 3=bed net is old and worn-out, 4= I have no bed net. 5= pack not opened, 6= other specify)* |  |  |  |  |  |  |  |  |  | |  |
| 49 | ***Who usually sleep under the net?*** *(1=father and mother, 2=mother and under five children, 3=pregnant woman, 4=children above 5, 5= all; 6= other ______ )* |  |  |  |  |  |  |  |  |  | |  |

|  | ***Prevention and control practices*** | Attributes | | | | | | | | | |
| --- | --- | --- | --- | --- | --- | --- | --- | --- | --- | --- | --- |
|  |  | 1 | 2 | 3 | 4 | 5 | 6 | 7 | 8 | 9 | 10 |
| 50 | ***Do you wash nets regularly? (1=yes, 2=no)*** |  |  |  |  |  |  |  |  |  |  |
| 51 | ***If yes, how often do you wash your bed nets?*** *(1=every month, 2=every 2 month, 3=every 6 month, 4=once in a year, 5= I don’t know)* |  |  |  |  |  |  |  |  |  |  |
| 52 | ***Do you mend your bed net if they are teared? (1= yes, 2= no)*** |  |  |  |  |  |  |  |  |  |  |
| 53 | ***If no why? (1=net is not worn, 2=I was not informed, 3=other)*** |  |  |  |  |  |  |  |  |  |  |
| 54 | ***How many months are needed to plaster house after spraying? (1= I don’t know, 2= after 4to 6 months, 3= after 6 months*** |  |  |  |  |  |  |  |  |  |  |
| 55 | ***Do you involve in draining and filling small water bodies around your compound? (1=yes, 2=no)*** |  |  |  |  |  |  |  |  |  |  |
| 56 | ***If no, why? (1= no water body nearby, 2= I was not informed, 3=other)*** |  |  |  |  |  |  |  |  |  |  |
| 57 | ***Have you ever participated in community-based mosquito breeding source management? (1= yes, 2=no)*** |  |  |  |  |  |  |  |  |  |  |
| 58 | ***If no, why? (1= there was no camping, 2= there were no facilitators, 3= others*** |  |  |  |  |  |  |  |  |  |  |
| 59 | ***Do you believe that cleaning the environment is useful for malaria control? (1=yes, 2= no)*** |  |  |  |  |  |  |  |  |  |  |
| 60 | ***Do you discuss about malaria problem & its prevention with your family? (1=yes, 2=no)*** |  |  |  |  |  |  |  |  |  |  |
| 61 | ***Is there any malaria control committee/group in your village (1=yes, 2=no?)*** |  |  |  |  |  |  |  |  |  |  |
| 62 | ***Have you ever participated in community-based malaria control activities such as filling, draining? (1= yes, 2= no)*** |  |  |  |  |  |  |  |  |  |  |
| 63 | ***What is your contribution as community member in malaria control? (****1=I should report febrile cases immediately to HEW, 2=I should involve community wide events such as draining, filling, 3=I should stop creating water pools, 4= I should teach my family and my neighbors about malaria control, 5 = don’t know* |  |  |  |  |  |  |  |  |  |  |
| 64 | ***Have you ever received community-based malaria control education? (1=yes, 2=no)*** |  |  |  |  |  |  |  |  |  |  |
| 65 | ***If no why, (1=busy with farming activities, 2=there was no educator, 3=I don’t believe in educators, 4=others (specify_______________)*** |  |  |  |  |  |  |  |  |  |  |
